# Long standing cyanosis in congenital heart disease does not cause diffuse myocardial fibrosis

**DOI:** 10.1186/1532-429X-16-S1-P108

**Published:** 2014-01-16

**Authors:** Ahmed E Kharabish, Christian Meierhofer, Stefan Martinoff, Peter Ewert, Heiko Stern, Sohrab Fratz

**Affiliations:** 1 Pediatric Cardiology and Congenital Heart Defects, German Heart Center, Munich, Germany; 2 Radiology and Nuclear Medicine, German Heart Center, Munich, Germany

## Background

The assumption of the presence of diffuse myocardial fibrosis in long standing cyanotic congenital heart disease (CHD) inspired us to noninvasively determine the myocardial extracellular volume (ECV) using contrast CMR (T1 mapping).

## Methods

T1 maps were measured pre and 3-10 minutes after the infusion of 0.15 mmol/kg of gadolinium on 25 subjects. Seven adult patients with longstanding cyanotic CHD and no previous surgical history (aged 16-53 yrs and oxygen saturations of 69-90%), nine normal subjects (aged 14-49 yrs), and nine patients with previously cyanotic CHD after total repair during which a heart lung machine was used (aged 2 months-58 yrs). Images were obtained in a mid-ventricular short axis plane. Late gadolinium enhancement using the phase sensitive inversion recovery (PSIR) sequence was performed to exclude scar areas. The T1 values were measured in two areas of the myocardium, in the septum and in the left ventricular posterior or inferior wall, such that same areas were assessed in every patient in the pre and post contrast T1 scan. ECV was calculated according to (1-hematocrit)*(ΔR1 myocardium/ΔR1 blood).

## Results

Patients with cyanosis had significantly lower ECV percentage than the previously cyanotic patients after total repair (septum: 22 ± 2% vs 35 ± 12%, p = 0.01; LV wall: 22 ± 2% vs 30 ± 7%, p = 0.02, respectively). No significant differences were found between patients with cyanosis and normal controls (septum: 22 ± 2% vs 24 ± 1%, p = 0.14; LV wall: 22 ± 2% vs 24 ± 2%, p = 0.21, respectively). ECV values were significantly different between the three groups in both septum and LV wall (f = 0.007 and f = 0.02, respectively).

## Conclusions

Long standing cyanosis in congenital heart disease without cardiac surgery does not cause diffuse myocardial fibrosis or expansion of the myocardial extra cellular volume.

## Funding

All authors have no conflict of interest.

